# Supplementary material for: A matter of size: Comparing IV and OLS estimates
Source: PLoS One. 2025 Oct 22;20(10):e0334392. doi: 10.1371/journal.pone.0334392 (PMC12543180; doi:10.1371/journal.pone.0334392)
Supplement: S1 Appendix — See Sects A–C and Table A.1. (PDF) [file pone.0334392.s001.pdf]

A Matter of Size: Comparing IV and OLS estimates

Riccardo Ciacci ◕,

◕ Department of Economics, Universidad Pontificia Comillas, Spain, Madrid. Email: riccardo.ciacci@eui.eu

Keywords: Instrumental variables, comparison IV and OLS estimates, endogeneous variables.
JEL codes: C26

Abstract

Sizeable differences between OLS (Ordinary Least Squares) and IV (Instrumental Variables) estimates might be interpreted in the literature as evidence that the instrument is not valid. Yet, to the best of our knowledge, this comparison is carried out using only the OLS coefficient as a benchmark and does not account for statistical measures or information from the OLS regression. This paper establishes a framework where [1]'s methodology might be used to compare objectively OLS and IV estimates. This methodology offers evidence to support or discard IV estimates with respect to the OLS regression.

Appendix

A The psacalc command in this framework

This section discusses how to use the psacalc command in Stata. Download [1] Stata command typing: `ssc install psacalc`

A.1 Syntax

The syntax of this command is as follows: `psacalc estimate varname [,options]`
where `estimate` stands for either `beta` or `delta`. If we choose `beta`, we have to fix a value `#` for `delta` in options typing `,delta(#)`. Symmetrically, if we choose `delta`, we have to fix a value `#` for `beta` in options typing `,beta(#)`

A.2 Description

`psacalc` estimates either `beta` (resp. `delta`) fixing a value for `delta` (resp. `beta`). `psacalc` is a post-estimation command that can be run after a linear regression model. Namely, the linear regression model commands that might be used are: `areg`, `xtreg` or `regeress`.

A.2.1 psacalc beta vs psacalc delta

`psacalc` computes either estimates of treatment effects or the relative degree of selection for an independent variable `varname` in a linear model as introduced in [1]. These estimators are consistent assuming that selection on the observables is proportional to selection on unobservables.

psacalc beta

`psacalc beta varname, delta(#)` implies the command computes the implied treatment effect `beta` given a chosen value `#` of the coefficient of proportionality between unobservables and observables `delta(#)` chosen. If there are multiple solutions, by default the command chooses the solution that minimizes the squared difference to the estimated treatment effect in the controlled regression and without changing the direction of the bias (assumption 3 in [1]).
In the setting considered in this paper, one needs to fix `delta` to check how large such coefficient needs to be in a certain linear model to bring about `beta` to compare it to the IV estimated coefficient β_{IV}. In our setting, it is key to establish the sign of `delta` given the observables considered in the linear regression.

psacalc delta

`psacalc delta varname, beta(#)` implies the command computes the relative degree of proportionality between unobservables and observables `delta` associated to the chosen value `#` of the treatment effect `beta(#)` chosen.
In the setting considered in this paper, `beta` matches an IV estimate `beta(`β_{IV}`)` to check how large `delta`, of the determined sign, needs to be to bring about such estimates in a linear model.

A.3 Options

A.3.1 Main options

`mcontrols(varlist)` observable controls included in this list are left out from the set of observables proportionally related to unobservables. Put it differently, these observables are assumed to be unrelated to unobservables.
`rmax(\#)` this option specifies the R_{max} which would result in a theoretical scenario in which all relavant unobservables were included in the linear model. If not specified, it is set to 1 by default.

A.3.2 Further options

`model` specifies a model to be estimated to run the command. This option is used when `psacalc` is used as a stand-alone command instead of a post-estimation command (i.e. after a linear model). This further option is useful when obtaining bootstrap estimates of the standard errors of beta and delta. Three types of models are supported: `areg`, `xtreg` or `regression`.

A.3.3 Saved results

Following the psacalc help file, this command saves the following results:

```
psacalc saves the following in r():

Scalars
r(delta)            proportional degree of selection
r(beta)             treatment effect

Macros
r(cmd)              command used for estimation
r(depvar)           dependent variable
r(indepvars)        independent variables
r(treatment)        treatment variable
r(mcontrol)         unrelated controls
r(type)             type of calculation, beta or delta

psacalc beta saves the following in r():

Scalars
r(dist)             squared difference from treatment effect to
                            controlled beta,

                     (beta-beta_tilde)^2

r(root_count)       number of solutions
r(altsol1)          alternative solution 1
r(altdist1)         squared difference of alternative solution 1 to
                            controlled beta
r(altsol2)          alternative solution 2
r(altdist2)         squared difference of alternative solution 2 to
                            controlled beta
```

B Example

This section discusses how to compute estimates as the ones in the Section Empirical validation using Stata.

B.1 Table 6

Download data from *https://economics.mit.edu/faculty/acemoglu/data/ajr2001*. This example reproduces Table 6 which is based on the IV regression of Column (7) of Table 4 of [16].
Load the data: `use maketable4, clear`
Create the *other continent* variable following authors' readme file

```
gen other_cont=.
replace other_cont=1 if (shortnam=="AUS" | shortnam=="MLT" | shortnam=="NZL")
recode other_cont (.=0)
tab other_cont
```

Generate a global with the values that `delta` will take:

```
gl list 1 2 3 4 5 10 20 50 100 1000
```

Run the OLS regression and then for each value run a `psacalc beta  avexpr, delta(#)` command to estimate the corresponding `beta`. Namely, using the OLS regression corresponding to such estimates the code is as follows:

```
reg logpgp95  avexpr  africa asia other_cont if baseco==1, rob
forv i=1/10{
local a : word `i' of $list
psacalc beta avexpr, delta(-`a')
}
```

This command computes `beta` for each specified value `#` of `delta`. As an example for the following command `psacalc beta  avexpr, delta(-1)` the output is as follows:

```
---- Treatment Effect Estimate ----
|     Estimate           Sq. difference      Bias changes
|                      from controlled beta    direction
-------------+---------------------------------------------------------------
Beta         |       0.62032                    .
Alt. sol. 1  |            .                     .
Alt. sol. 2  |            .                     .
-------------+---------------------------------------------------------------

---- Inputs from Regressions ----
|      Coeff.            R-Squared
-------------+---------------------------------------------------------------
Uncontrolled |        0.52211         0.540
Controlled   |        0.42383         0.704
-------------+---------------------------------------------------------------

---- Other Inputs ----
-------------+---------------------------------------------------------------
R_max        |   1.000
```

```
Delta        |   -1.000
Unr. Controls|
-------------+----------------------------------------------------------
```

For each `psacalc beta  avexpr, delta(#)` the output displays an estimate `beta` and alternative solutions if there are any. Moreover, it displays the estimated coefficients and R^2 of the controlled and uncontrolled OLS regression. The output also shows the R_{max} and `delta` used.
Finally, run the IV regression to save the IV estimates.

```
ivreg logpgp95 (avexpr=logem4)  africa asia other_cont if baseco==1, first
```

B.2 Figure

This section guides the reader to produce a graph plotting either the upper bounds or the lower bounds of `beta` associated to different values of `delta`. After establishing the sign of `delta` run `psacalc delta varname, beta(#)` to know which values to assign to `delta` when building the graph. For example, in the case considered in Section Example 2 this would be `psacalc delta sex_purchase_abs_, beta(0.0189)`.
Generate missing values variables for two objects to plot:

```
g delta=.
g est_bet=.
```

Replace them with the chosen value `i` of `delta` and the estimated bound of the treatment effect `beta`, from line 1 onwards. Specifically, the code is as follows:

```
psacalc beta sex_purchase_abs_, delta(i)
replace delta=i in 1
replace est_bet=r(beta) in 1
```

Run the corresponding IV regression, then save the estimated coefficient and make a graph with the estimated `beta` associated to each `delta` and a vertical line for the IV estimated coefficient. Namely, after run the IV regression save the results and plot the variables previously estimated.

```
mat def b=e(b)
gl b1=el(b,1,1)

scatter  delta est_bet if delta!=. , c(l) xli($b1) yti("Delta")  ///
xti("Estimated coefficient, upper-bound identified set")
        graphregion(color(white))
```

C Univariate and multivariate cases

Univariate

Let the PRF be equation (1). However, given the nature of w we can only run equation (2).
Consider $\widehat{\beta}_2 = (D'M_xD)^{-1}(D'M_xY)$, where $M_x \equiv I - X(X'X)^{-1}X'$.
Then using equation (1) we get:

$$\widehat{\beta}_2 = \beta_1 + \gamma \frac{d'_{ih}M_x w_{ih}}{d'_{ih}M_x d_{ih}} \tag{A.1}$$

Hence, expanding M_x in the univariate case we obtain:

$$\text{plim } \widehat{\beta}_2 = \beta_1 + \gamma \frac{\delta Cov\,(d_{ih}, X_{ih})\, Var(w_{ih}) - Cov(d_{ih}, x_{ih}) Cov(X_{ih}, w_{ih})}{Var(d_{ih}) Var(X_{ih}) - Cov(d_{ih}, X_{ih})^2} \tag{A.2}$$

We can rearrange equation (A.2) using equation (3) to get:

$$\delta = \frac{\widehat{\beta}_2 - \beta_1}{\gamma} \frac{Var(d_{ih}) Var(x_{ih}) - Cov(d_{ih}, X_{ih})^2}{Cov(d_{ih}, X_{ih}) Var(w_{ih}) - Cov(d_{ih}, X_{ih}) Cov(X_{ih}, w_{ih})} \tag{A.3}$$

which is similar to equation (5) of the univariate case.

Multivariate

Note that regression model (1) is equivalent to:

$$\begin{bmatrix} y_1 \\ \vdots \\ y_n \end{bmatrix} = \begin{bmatrix} d_1 \\ \vdots \\ d_n \end{bmatrix} \beta_1 + \begin{bmatrix} w_1 \\ \vdots \\ w_n \end{bmatrix} \gamma + \begin{bmatrix} x_{11} & \cdots & x_{1k} \\ \vdots & \ddots & \vdots \\ x_{n1} & \cdots & x_{nk} \end{bmatrix} \begin{bmatrix} \theta_1 \\ \vdots \\ \theta_n \end{bmatrix} + \begin{bmatrix} \varepsilon_1 \\ \vdots \\ \varepsilon_n \end{bmatrix}$$

In compact form equivalent to:

$$Y_{ih} \ = D_{ih}\beta_1 \ + \ W_{ih}\gamma \ + \ X_{ih}\theta_1 \ + \ \varepsilon_{1ih} \tag{A.4}$$

Yet, given the nature of w we can only run:

$$Y_{ih} \ = D_{ih}\beta_2 \ + \ X_{ih}\theta_2 \ + \ \varepsilon_{2ih} \tag{A.5}$$

To this extent, note that:

- Σ_{DX} (1 x k): is the cross covariance matrix between D and X
- Σ_{DW} (1 x 1): is the covariance matrix between D and W, given that both of them have dimension $k = 1$, it is equivalent to the scalar $Cov(d_{ih}, w_{ih})$.
- Σ_D (1 x 1): is the variance covariance matrix of D, for the same reason equivalent to the scalar $Var(d_{ih})$ in this setting.
- Σ_X (k x k): is the variance covariance matrix of X.
- Σ_X^* (1 x k): is a row-vector containing the diagonal of the variance covariance matrix of X.
- Σ_W (1 x 1): is the variance covariance matrix of W, for the reasons expounded above it is equivalent to the scalar $Var(w_{ih})$ in this setting.
- Σ_{XW} (k x 1): is the cross covariance matrix between X and W

Consider $\widehat{\beta}_2 = (D' M_x D)^{-1}(D' M_x Y)$, then using equation (A.4) we get:

$$\widehat{\beta}_2 = \beta_1 + \gamma(D' M_x D)^{-1}(D' M_x W) \tag{A.6}$$

where $M_x \equiv I - X(X'X)^{-1}X'$
In the multivariate case, equation (3) becomes:

$$\delta \Sigma_{DX} (\Sigma_X^*)'^{-1} = \Sigma_{DW} \Sigma_W^{-1} \tag{A.7}$$

Note that by $(\Sigma_X^*)'^{-1}$ we denote a column vector containing all the values of Σ_X^* each one of them elevated to -1.

Then, expanding M_x in equation (A.6), we get:

$$\text{plim}\,\widehat{\beta}_2 = \beta_1 + \gamma(\Sigma_D - \Sigma_{DX}\Sigma_X^{-1}\Sigma_{DX}')^{-1}(\Sigma_{DW} - \Sigma_{DX}\Sigma_X^{-1}\Sigma_{XW}) \tag{A.8}$$

Combining with (A.7) we obtain:

$$\delta = \left[(\Sigma_D - \Sigma_{DX}\Sigma_X^{-1}\Sigma_{DX}')\gamma^{-1}(\widehat{\beta}_2 - \beta_1) + \Sigma_{DX}\Sigma_X^{-1}\Sigma_{XW}\right]\Sigma_W^{-1}(\Sigma_X^*)'\Sigma_{DX} \tag{A.9}$$

This could be extended easily to cases where W has dimension k but given W is an unobservable it would not change the results or their interpretation (i.e. Σ_{DW} would have dimension (1 x k) and we would need to define Σ_W^* as it was done for X)

Table A.1. Summary: Multivariate Case

Symbol	Meaning	Role / Assumption
Y_{ih}	Outcome vector	$n \times 1$ vector of outcomes
D_{ih}	Treatment vector	$n \times 1$ vector of treatments
X_{ih}	Observed controls	$n \times k$ matrix of observed covariates
W_{ih}	Unobserved controls	$n \times 1$ vector of unobserved covariates
β_1	True treatment effect	Scalar causal effect
θ_1	Effect of observed controls	$k \times 1$ vector of coefficients
γ	Effect of unobservable	Scalar coefficient of W_{ih}
Σ_{DX}	Covariance: D and X	$1 \times k$ cross-covariance matrix
Σ_{DW}	Covariance: D and W	Scalar (since W is univariate)
Σ_{XW}	Covariance: X and W	$k \times 1$ cross-covariance vector
Σ_D	Variance of D	Scalar
Σ_X	Variance of X	$k \times k$ matrix
Σ_W	Variance of W	Scalar
Σ_X^*	Diagonal of Σ_X	$1 \times k$ row vector
δ	Coefficient of proportionality	Scalar; measures relative selection on observables vs. unobservables

Acknowledgments

I am deeply grateful to Juan José Dolado and five referees for their insightful comments on the results of this paper. Any remaining errors are, of course, my sole responsibility.

References

1. Oster E. Unobservable selection and coefficient stability: Theory and evidence. Journal of Business & Economic Statistics. 2019;37(2):187–204.

2. Angrist JD, Krueger AB. Instrumental variables and the search for identification: From supply and demand to natural experiments. Journal of Economic Perspectives. 2001;15(4):69–85.

3. Angrist JD, Pischke JS. Mostly harmless econometrics: An empiricist's companion. Princeton University Press; 2008.

4. Imbens GW. Sensitivity to exogeneity assumptions in program evaluation. American Economic Review. 2003;93(2):126–132.

5. Altonji JG, Elder TE, Taber CR. Selection on observed and unobserved variables: Assessing the effectiveness of Catholic schools. Journal of Political Economy. 2005;113(1):151–184.
6. Alesina A, Giuliano P, Nunn N. On the origins of gender roles: Women and the plough. The Quarterly Journal of Economics. 2013;128(2):469–530.
7. Mejia D, Restrepo P. Bushes and bullets: illegal cocaine markets and violence in Colombia. Documento CEDE. 2013;(2013-53).
8. Bhuller M, Dahl GB, Løken KV, Mogstad M. Incarceration, recidivism, and employment. Journal of Political Economy. 2020;128(4):1269–1324.
9. Liu S. Incarceration of African American Men and the Impacts on Women and Children; 2020.
10. Murphy KM, Topel RH. Efficiency wages reconsidered: Theory and evidence. In: Advances in the Theory and Measurement of Unemployment. Springer; 1990. p. 204–240.
11. Altonji JG, Elder TE, Taber CR. An evaluation of instrumental variable strategies for estimating the effects of catholic schooling. Journal of Human resources. 2005;40(4):791–821.
12. Altonji JG, Conley T, Elder TE, Taber CR. Methods for using selection on observed variables to address selection on unobserved variables. Citeseer. 2010;.
13. De Luca G, Magnus JR, Peracchi F. Comments on Unobservable Selection and Coefficient Stability: Theory and Evidence and Poorly Measured Confounders are More Useful on the Left Than on the Right. Journal of Business & Economic Statistics. 2019;37(2):217–222.
14. Angrist J, Imbens G. Identification and estimation of local average treatment effects. Cambridge, Mass., USA: National Bureau of Economic Research; 1995.
15. Masten MA, Poirier A. Salvaging falsified instrumental variable models. Econometrica. 2021;89(3):1449–1469.
16. Acemoglu D, Johnson S, Robinson JA. The colonial origins of comparative development: An empirical investigation. American Economic Review. 2001;91(5):1369–1401.
17. Gallup JL, Sachs JD, Mellinger AD. Geography and economic development. International Regional Science Review. 1999;22(2):179–232.
18. Hall RE, Jones CI. Why do some countries produce so much more output per worker than others? The Quarterly Journal of Economics. 1999;114(1):83–116.
19. Ciacci R. Banning the purchase of sex increases cases of rape: Evidence from Sweden. Journal of Population Economics. 2024;37.
20. Farley M, Barkan H. Prostitution, violence, and posttraumatic stress disorder. Women & health. 1998;27(3):37–49.
21. Farley M, Cotton A, Lynne J, Zumbeck S, Spiwak F, Reyes ME, et al. Prostitution and trafficking in nine countries: An update on violence and posttraumatic stress disorder. Journal of trauma practice. 2004;2(3-4):33–74.
