# Supplementary material for: A matter of size: Comparing IV and OLS estimates
Source: PLoS One. 2025 Oct 22;20(10):e0334392. doi: 10.1371/journal.pone.0334392 (PMC12543180; doi:10.1371/journal.pone.0334392)
Supplement: Attachment — Submitted filename: Evaluation_Report_PONE-D-24-23558.pdf [file pone.0334392.s002.pdf]

Evaluation Report

(PONE-D-24-23558)

A Matter of Size: Comparing IV and OLS estimates

The submitted work addresses the discrepancies between Ordinary Least Squares (OLS) and Instrumental Variables (IV) estimates which are often seen as a red flag, suggesting potential issues with the validity of the instrument. Traditionally, these differences have been evaluated by directly comparing the OLS and IV coefficients, with little consideration given to the statistical information underlying the OLS estimates. This article introduces an approach by utilizing Oster's (2019) methodology to provide a more rigorous and objective framework for comparing OLS and IV estimates. A few examples, along with an extensive simulation study, validate the proposal. Therefore, this article holds a significant merit and should be considered for publication.

However, there is a minor observation: why did the author choose a panel data model for the presented framework?

(End of evaluation)
