# Supplementary material for: A matter of size: Comparing IV and OLS estimates
Source: PLoS One. 2025 Oct 22;20(10):e0334392. doi: 10.1371/journal.pone.0334392 (PMC12543180; doi:10.1371/journal.pone.0334392)
Supplement: Attachment — Submitted filename: Evaluation_Report_PONE-D-24-23558R1.pdf [file pone.0334392.s004.pdf]

Evaluation Report

(PONE-D-24-23558.R1)

A Matter of Size: Comparing IV and OLS estimates

The minor observation has been addressed and the article is fit for acceptance.

(End of evaluation)
