# Supplementary material for: A matter of size: Comparing IV and OLS estimates
Source: PLoS One. 2025 Oct 22;20(10):e0334392. doi: 10.1371/journal.pone.0334392 (PMC12543180; doi:10.1371/journal.pone.0334392)
Supplement: Attachment — Submitted filename: review.pdf [file pone.0334392.s009.pdf]

Title: ***A Matter of Size: Comparing IV and OLS estimates***
Manuscript ID: PONE-D-24-23558R3

This is a technically solid and methodologically focused paper that proposes an objective framework—based on Oster (2019)—to compare IV and OLS estimates. The motivation is clear: large differences between IV and OLS coefficients are often interpreted as evidence of instrument invalidity, but without a formal benchmark. By formalizing this comparison and illustrating it through both simulations and observational applications, the author fills a methodological gap in applied econometrics.

There are some issues that the authors need to address before the manuscript can be considered for publication. The following are my comments describing these issues.

1- Contribution and Relevance

- The contribution is methodological and addresses a gap in applied econometrics practice: a formal, reproducible approach for OLS–IV comparison.
- The integration with Oster's method is original in this context, and the emphasis on implementability (including Stata code) is a major strength.
- The simulations convincingly show that large IV/OLS ratios do not necessarily imply instrument invalidity.

2- Theoretical Framework

- The derivation from equations (1)–(5) is correct but dense. Some intermediate economic intuition would help applied readers, e.g., explaining in words:
  - Why the proportionality assumption is plausible in some settings.
  - What kinds of omitted variables w are captured by δ in applied terms.
- The paper moves quickly from the univariate to the multivariate setting; a table summarizing symbols, assumptions, and their roles would improve accessibility.

3- Assumption Discussion

- The proportional selection assumption (equation (3)) is strong. While the author mentions that prior knowledge of the setting is crucial, more discussion is needed on:
  - Situations where the assumption is likely violated (e.g., when observables are weak proxies for unobservables).
  - Sensitivity of results to mis-specification of Rmax.
  - Possible robustness checks (e.g., alternative Rmax values, bounding δ using prior studies).

4- Simulation Study

- The Monte Carlo section is informative, but the practical meaning of thresholds such as $|\delta|<1$ or $|\delta|<2$ is not fully explained. The reader would benefit from a short "practical guidelines" subsection translating these findings into applied advice (e.g., what value of $|\delta|$ should start to raise concern).

5- Empirical Applications

- **Acemoglu et al. (2001):**
  - The conclusion that no plausible δ can reconcile OLS and IV estimates is striking. The discussion could more explicitly connect this to historical debates on settler mortality and institutions to help contextualize why IV estimates may be inflated.
- **Ciacci (2024):**
  - This example benefits from a clear rationale for the instrument (flights), but robustness to alternative specifications or placebo outcomes would strengthen the case.
  - Since this is the author's own prior work, a note on possible self-selection in variable choice could be made transparent.

6- Interpretation and Limitations

- The paper is careful to state that large δ values could indicate either invalid instruments or heterogeneous effects. However:
  - A more structured decision tree could help applied researchers interpret results (e.g., "If δ is large, check X, Y, Z…").
  - The policy implications of such findings could be briefly discussed — this would broaden the readership beyond methodologists.
