# Supplementary material for: A matter of size: Comparing IV and OLS estimates
Source: PLoS One. 2025 Oct 22;20(10):e0334392. doi: 10.1371/journal.pone.0334392 (PMC12543180; doi:10.1371/journal.pone.0334392)
Supplement: Attachment — Submitted filename: Referee 2.rtf [file pone.0334392.s003.rtf]

Dear Reviewer, 

Many thanks for your comments that helped me improve the paper. I reckon there might have been a misunderstanding with the aim of my paper. To this extent, please let me know if the new version is clear on this matter, I can further clarify as you consider fit. The misunderstanding is due to the issue that my paper does not deal with weak instruments specifically nor with invalid instrument broadly. My paper suggests a technique to compare OLS and IV estimates that works if the instrument is valid. However, this methodology does not deal with validity of the instruments. Strong correlation of the instrument with the endogenous variable might be tested using the first stage coefficient while exogeneity of the instrument and the exclusion restriction need knowledge of the specific setting to provide pieces of evidence on the plausibility of each of these two assumptions. In settings where these three assumptions are considered plausible, the IV estimates and OLS estimates might be compared using my methodology. In this case, the sizes of OLS and IV estimates might compared using the delta introduced by Oster (2019). There is not statistical test of this delta. Still, computing its size is a way to check whether OLS and IV estimates differ substantially.  Please find below my replies (in bold) in a one to one basis.
Reviewer #2: Review report on PONE-2024-23558 “A Matter of Size: Comparing IV and OLS estimates”  In this paper, the author compares the significant differences between Ordinary Least Squares (OLS) and Instrumental Variables (IV) estimates, particularly in situations where the instrument(s) may be invalid. The framework of Oster’s (2019) methodology is also employed to contrast OLS and IV estimates. However, this article requires substantial revisions, as detailed below in the major and minor concerns. Major Comments: • The paper currently resembles a short report rather than a comprehensive academic study. It lacks sufficient detail on the topic of interest and the underlying motivation. For example, the primary objective of the paper should be clearly explained and linked to previous work. The author should clarify the research problem, identify the limitations of both the IV and OLS approaches, and outline what previous studies have accomplished in this area. Furthermore, the author’s contribution relative to existing studies should be explicitly stated in the introduction. Many thanks for this comment. I have addressed this in the article adding the references you suggest and highlighting the contribution. As for the most technical information (e.g. limitations of the approaches) I prefer not to have it run the introduction but I can write it there if you consider it suit.
• In Section 2, just after Equation (4), the statement “Recall that if the instrument is valid, the IV coefficient consistently estimates β1” - the author should theoretically define the test used to determine the validity of instruments and report the type I and II error rates in the simulation results. Additionally, the concepts of “valid” and “invalid” instruments should be clearly defined, along with the potential consequences of using invalid instruments. Many thanks for this observation. I think there has been a misunderstanding here. I do not suggest any test I merely state that if the instrument is valid that estimator is consistent. As for the definitions of valid and invalid instruments they are the standard definitions used in the literature. However, if you have any particular definition that might clarify some doubts you encountered please let me know and I will add them.
• The theoretical motivation for comparing OLS and IV estimates could be strengthened. The author should discuss the asymptotic properties of OLS and IV within the framework provided by Oster (2019). • While the paper reports the point estimation of δ, it is unclear how the author tests the significance of δ. This aspect needs to be addressed. I understand this comment, the issue is that I do not test the significance of δ. I merely provide a framework where one can compute delta and some boundaries to discuss but this is not a statistical test. To this extent, there are not distribution assumptions but I understand this might be a limitation to my paper (and it also offers space for further research on this issue, an easy contribution would be to suggest a statistical test for δ, yet in this direction more assumptions are needed.). 
• The paper should explore the behavior of the IV/OLS ratio when the instruments are weak. It would be interesting to include results for scenarios involving both strong and weak instruments in the simulation.
Thanks for this comment but this article does not deal directly with weak instruments. This study provides a framework to compare OLS and IV estimates once we assume instruments are valid. Yet if instruments are not valid (that is, either not strongly correlated with endogenous regressor, exogenous or exclusion restriction is not met; any of these three conditions suffices to have an invalid instrument, while only the first one implies a weak instrument) this article does not offer any solution. The research question about how to compare OLS and IV estimates if instruments are weak or if instruments are invalid is a legitimate and interesting one yet it falls outside the scope of this article.
 Additionally, since real-world experiments often involve small sample sizes, I suggest that the author conduct simulations with smaller samples, starting with a sample size of 1000. Another simulation experiment could be added to explore different model specifications, including linear, nonlinear, and semiparametric models. Also, report the bias and RMSE. Many thanks for this comment. The issue is that this methodology relies on a large number of observations in order to be used. In settings where the number of observation is low it is better not to rely on this methodology. 
Minor Comments: • Although OLS and IV are common abbreviations, it would be helpful to spell them out in the abstract. Done
• The objective of the paper is not clearly articulated. If the first paragraph is intended to provide the motivation—stating that "Nonetheless, in the literature, to the best of my knowledge, there is no formal methodology to compare these two estimates"—then I must point out that the validity of instruments has been widely discussed in the literature. Many studies have compared the performance of OLS and Two-Stage Least Squares (TSLS), such as Murray (2006), Kolesár et al. (2015), and DiTraglia (2016), among others. This article does not deal with validity of instruments. An instrument is valid if it is strongly correlated to the endogenous variable, it is exogenous and it only affects the outcome variable through the endogenous variable (exclusion restriction). This paper does not deal with any of these three assumptions. It takes them for granted and it adds to the literature a way to compare OLS and IV estimates once we believe the instrument is valid. I have re-formulated this part of the first paragraph to clarify this issue. I am sorry it was not clear. Any further edit you consider fit please let me know.
• The differences between Equation (1) and Equation (2) are not clear, as both seem to involve the same dependent variable yih. It appears that these models are intended to illustrate omitted variable bias. The author should clarify each model for the reader's benefit. There are different notations for the parameters (α1, α2, β1, β2, etc.), but not for the treatment variable and observed controls, which should be consistent. I have modified the lines. Please note that the betas are for the treatment, gamma for the unobserved variable and theta for the controls. I can modify it further as you consider best.
• The author should clarify whether Model (1) is a reduced form model. Additionally, the first-stage regression and the instruments used in the model should be explicitly defined. Ideally equation (1) is a reduced form regression but it cannot be run because omega is unobserved. Clearly once omega is not included in the model specification (1) is not a reduced form anymore due to the omission of that very variable. I wanted to keep the analysis as simple as possible. Thus, I did not include the first-stage since I would not write anything about it, only describing each variable. Yet, if you want me to include the first-stage equation please tell me in what section you would include it and exact part of the text please.
• In Section 3.1, the data-generating process for the simulation experiment should be clearly described. The author should specify the distribution of the treatment effect. Sure. I have added the do file to replicate the computations and a couples of lines in the document telling the reader about the availability of such a do file.
• Sections 4 and 5 should be moved to an online supplementary materials section, as they may not be essential to the main body of the paper. Done 
Oster, E. (2019). Unobservable selection and coefficient stability: Theory and evidence. Journal of Business & Economic Statistics 37(2), 187–204. Murray, M. P. (2006). Avoiding invalid instruments and coping with weak instruments. Journal of economic Perspectives, 20(4), 111-132. Kolesár, M., Chetty, R., Friedman, J., Glaeser, E., & Imbens, G. W. (2015). Identification and inference with many invalid instruments. Journal of Business & Economic Statistics, 33(4), 474-484. DiTraglia, F. J. (2016). Using invalid instruments on purpose: Focused moment selection and averaging for GMM. Journal of Econometrics, 195(2), 187-208.
