# Supplementary material for: A matter of size: Comparing IV and OLS estimates
Source: PLoS One. 2025 Oct 22;20(10):e0334392. doi: 10.1371/journal.pone.0334392 (PMC12543180; doi:10.1371/journal.pone.0334392)
Supplement: Attachment — Submitted filename: reviewer comments_reply.docx [file pone.0334392.s006.docx]

**A Matter of Size: Comparing IV and OLS estimates**

**Manuscript Number: PONE-D-24-23558**

**Reviewer Comments**

**Please find my comments in red in a point-by-point basis**

This is a revision of the revised version of the paper which addresses the comments by the reviewers to the first version. I beg to agree with most comments by reviewer #2 and it is my view that the revised version did not address them in a satisfactory manner. *My recommendation is not to publish the paper* on the following grounds:

- There is no doubt that the comparison of OLS and IV estimates is an important issue when someone may be discussing the robustness of estimates of treatment effects.

Many of the comments written by this reviewer are “empty comments” as this one. They are empty because they merely highlight an important point in the literature, but they do not say anything about my paper.

- The purpose of the article is not clearly defined and the findings in the literature should be acknowledged, in particular Oster (2019). This paper is an implementation of the ideas in Oster (2019) and as such does not add anything new to current knowledge.

This is not true. The aim is my paper is to adapt Oster (2019) paper to OLS and IV coefficient comparison. In Oster (2019) it is never mentioned the word IV. That paper is not about IV broadly and least it is not about comparing OLS and IV coefficients. That paper is about comparing OLS coefficients after including control variables.

- The paper provides examples for the implementation of the second way robustness statements might be made (i.e. making statements about $R_{max}$, (Oster, 2018, p. 196-197). I couldn’t locate the contributions the author claims to have added to the text. If the paper is an application of Oster’s ideas, at least the links with Imbens (2003) and Altonji, Elder & Taber (2005) should be described.

To this extent, please check my reply to reviewer 2. I have added the literature she/he suggested. Furthermore, in the draft I submitted all the changes were written in red so that the reader could easily find them.

- The issue of evaluating the robustness of results is intrinsically empirical. It is assumed that when designing a study, the researchers make decisions about what is an appropriate size for the treatment effect, how to control for confounders and which covariates should be included. Both theory and experience play an important role at this stage. Some of the confounders and covariates might not be observable at the time the study is being designed, and early decisions are made on how to deal with this problem (i.e. instrumental variables or any other approach). This brings me to the topic of the paper, which is how to assess the magnitude of biases due to unobserved variables and likely measurement errors on the estimation of treatment effects. A well-designed research would determine the size of the proportionality coefficient and the validity of candidates instruments in advance. Common sense suggests that the contribution of instruments to selection should be of a lower magnitude than that from the controls. This leave us with a desired value of 1 for the proportionality coefficient.

Again, this comment is “empty”, it does not say anything about my article. I also would like to emphasize that the aim of my paper is to remove “common sense” comparisons based on eyeballing the OLS and IV coefficients. The methodology I introduce allows scholars to compare those two coefficients stating exactly the assumptions they make for such a comparison.

- Most evaluations in the literature use quasi-experimental designs or are designed as observational studies. They use instruments as part of an identification strategy to introduce enough variability in the estimation of the treatment effects. Decisions on what a valid set of instruments should be, form part of the design process.

Another “empty comment”

- The paper does not include information on the context in which the Oster´s method is being applied. The point that the validity of instruments is a crucial assumption is something that needed to be stressed early in the introduction.

This comment highlights the reviewer does not know IV techniques. Using IV always implies believing the instrument is valid. Indeed, there are a set of solutions whenever the scholar believes the instrument is not valid. Accordingly, if a scholar believes the instrument is invalid it would not make sense to compare the IV estimates given by that invalid instrument to the OLS estimates.

- Later in the methodological section, there is continuous reference to the fact that the proportionality coefficient may indicate that the instruments might not be valid, a matter that causes confusion when reading the paper.

The paper is clear on this issue. If the coefficient of proportionality is relatively high then the scholar knows there might be different issues at stake and further research is needed on this. As I write in page 3 “Since this analysis takes into account inclusion of controls, size of variances and movement of R2, among other things, large values of   might imply either that the instrument is not valid or that there are heterogeneous effects and the IV estimates are estimating them for a subpopulation [6]. In this circumstance (i.e. a large coefficient of proportionality) to distinguish between the two cases this methodology might be complemented using [17] methodology and carrying out an analysis, as in [10], to explore whether in the data there is empirical evidence supporting the IV is estimating heterogeneous effects for a subpopulation.”

- Before submission, the paper should have been reviewed by someone whose first language is English. There are lots of language problems.

Reviewer 1 did not find any of them and what it is more astonishing is that this was the second round of review. Language issues are raised in the first round and considered a minor point. Yet, I am afraid the main issue of this reviewer is not related to English but to lsck of understanding of the IV framework.
