# Supplementary material for: A matter of size: Comparing IV and OLS estimates
Source: PLoS One. 2025 Oct 22;20(10):e0334392. doi: 10.1371/journal.pone.0334392 (PMC12543180; doi:10.1371/journal.pone.0334392)
Supplement: Attachment — Submitted filename: reviewer comments_second_revision.docx [file pone.0334392.s007.docx]

**A Matter of Size: Comparing IV and OLS estimates**

**Manuscript Number: PONE-D-24-23558_R2_reviewer**

**Reviewer Comments**

This is a second revision to the revised version of the paper which addresses the comments by the reviewers to the first version. I must clarify that my comments to the previous submission (Manuscript Number: PONE-D-24-23558) were entirely based on the comments by reviewer #2, therefore my recommendation was not publishing it. I must say that rather disqualifying my comments for being “empty” or showing lack of “understanding of the IV methodology” the author could have taken them as useful suggestions to improve the paper. In what follows, I will expand on each of the comments I provided in my previous review aiming the author would take them seriously. I think that the research reported in the paper contributes to the literature on comparing IV and OLS estimates, under the assumption that the instruments are valid. My current comments refer to my previous ones but relate to the Revised Manuscript with Track Changes attached to the files received from PLOS ONE.

Since the changes I suggest are easy to implement, *my recommendation is to accept the paper for publication subject to making the changes suggested below*.

- There is no doubt that the comparison of OLS and IV estimates is an important issue when someone may be discussing the robustness of estimates of treatment effects.
  - The author qualified this comment as “empty” for not saying anything about the paper.
  - Perhaps, he might like to consider rewriting para 4 of the introduction along the following lines: “*Comparison of OLS and IV estimates is an important issue when discussing the robustness of estimates of treatment or intervention effects when researchers use observables to assess the bias generated by unobservable variables in OLS settings (Murphy and Topel 1990; ….; De Luca et. Al. 2019). The main contribution of this paper is to provide a formal methodology to make such a comparison by extending that developed in Oster (2019). Previous research has addressed the issue but lacks methodological support (Alesina et. al. 2013; ...;Liu 2020).*”
  - I strongly suggest to eliminate the reference to a “third contribution” as it contradicts the key assumption of instrument validity. It may confuse readers and distract them from the main purpose of the paper.
  - **I suggest to stress the point that the proposed method is not aimed at assessing the validity of the instruments**.
- The purpose of the article is not clearly defined and the findings in the literature should be acknowledged, in particular Oster (2019). This paper is an implementation of the ideas in Oster (2019) and as such does not add anything new to current knowledge.
  - The version of the Revised Manuscript with Track Changes addressed this issue, at least partially.
  - The author might like to consider **a more detailed elaboration on the extension to the case of *comparing OLS and IV estimates*. Oster´s method deals with the issue of unobserved controls but instrumental variables act like unobserved controls by allowing researchers to isolate the causal effect of a variable of interest**.
- The paper provides examples for the implementation of the second way robustness statements might be made (i.e. making statements about $R_{max}$, (Oster, 2018, p. 196-197). I couldn’t locate the contributions the author claims to have added to the text. If the paper is an application of Oster’s ideas, at least the links with Imbens (2003) and Altonji, Elder & Taber (2005) should be described.
  - Altonji, Elder & Taber (2005) (AET) was added to the list of references but not Imbens (2003) (American Economic Review,93: 126-132).
  - The author might like to mention somewhere in the introduction how Oster´s method relates to Imbens (2003) deals with the closely related logic behind partial least squares and AET proposed a method for evaluation of robustness under the assumption that the relationship between treatment and unobservables can be recovered from the relationship between treatment and observables.
- The issue of evaluating the robustness of results is intrinsically empirical. It is assumed that when designing a study, the researchers make decisions about what is an appropriate size for the treatment effect, how to control for confounders and which covariates should be included. Both theory and experience play an important role at this stage. Some of the confounders and covariates might not be observable at the time the study is being designed, and early decisions are made on how to deal with this problem (i.e. instrumental variables or any other approach). This brings me to the topic of the paper, which is how to assess the magnitude of biases due to unobserved variables and likely measurement errors on the estimation of treatment effects. A well-designed research would determine the size of the proportionality coefficient and the validity of candidates instruments in advance. Common sense suggests that the contribution of instruments to selection should be of a lower magnitude than that from the controls. This leave us with a desired value of 1 for the proportionality coefficient.
  - I regret the author did qualify this comment as “empty”. At least he might like to take some of the ideas above to provide some background to the response that the aim of this paper is to remove “common sense”.
- Most evaluations in the literature use quasi-experimental designs or are designed as observational studies. They use instruments as part of an identification strategy to introduce enough variability in the estimation of the treatment effects. Decisions on what a valid set of instruments should be, form part of the design process.
  - Again, the author considered this as an “empty” comment. The proposed method rests on the assumption of instrument validity and the paper shows how to implement it with panel data (Observational data ,Examples 1 and 2).
  - I suggest the author to include a text similar to that in the comment as background for the referred section. Keeping in mind that PLOS ONE is not an econometrics-economics journal and that the potential readers might not be familiar with modern micro econometrics approaches, a sort of an introduction to this section will provide a reason for the examples beyond the “complexity” in the data.
- The paper does not include information on the context in which the Oster´s method is being applied. The point that the validity of instruments is a crucial assumption is something that needed to be stressed early in the introduction.
  - The author replied by arguing about my lack of knowledge of IV techniques.
  - Both of us know that instruments may not be valid and that an IV analysis often only estimates the “local treatment effect” which may not generalize to the entire population.
  - Despite the author´s disqualification of this reviewer econometrics credentials, the issue addressed by this comment was stated at the end of the first paragraph of the introduction.
  - What would happen when this is not the case?
- Later in the methodological section, there is continuous reference to the fact that the proportionality coefficient may indicate that the instruments might not be valid, a matter that causes confusion when reading the paper.
  - This issue was addressed by reviewer #2 and I believe that it continues to be a weakness of the paper.
  - Since your method provides a point value for delta and has not distributional properties associated to it, how can you assess whether its value is large enough to indicate either the invalidity of the instrument, or heterogenous effects, or both?
- Before submission, the paper should have been reviewed by someone whose first language is English. There are lots of language problems.
  - My comment was based on PLOS ONE criterion 1 for publication: “PLOS ONE does not copyedit accepted manuscripts, so the language in submitted articles must be clear, correct, and unambiguous. We may reject papers that do not meet these standards. If the language of a paper is difficult to understand or includes many errors, we may recommend that authors seek independent editorial help before submitting a revision. These services can be found on the web using search terms like “scientific editing service” or “manuscript editing service.””
  - It is not a bad idea to check the quality of the writing with an expert.
