# Supplementary material for: A matter of size: Comparing IV and OLS estimates
Source: PLoS One. 2025 Oct 22;20(10):e0334392. doi: 10.1371/journal.pone.0334392 (PMC12543180; doi:10.1371/journal.pone.0334392)
Supplement: Attachment — Submitted filename: Referee 4 review_reply.docx [file pone.0334392.s010.docx]

I am deeply grateful to the referee for their thorough, insightful, and remarkably constructive feedback. Their opening statement—that the paper is "technically solid and methodologically focused" and successfully "fills a methodological gap"—provided tremendous encouragement. I especially wish to thank them for highlighting what they see as the core strengths of the manuscript: the clarity of its motivation, the originality of integrating Oster’s method into an IV framework, and the practical value of the provided Stata code, which they rightly identify as crucial for adoptability. The referee’s praise is greatly appreciated, and their detailed comments have been instrumental in refining the arguments and improving the overall quality of the paper. Please find my replies in red in a point by point basis.

**Title: *A Matter of Size: Comparing IV and OLS estimates* Manuscript ID: PONE-D-24-23558R3**

This is a technically solid and methodologically focused paper that proposes an objective framework—based on Oster (2019)—to compare IV and OLS estimates. The motivation is clear: large differences between IV and OLS coefficients are often interpreted as evidence of instrument invalidity, but without a formal benchmark. By formalizing this comparison and illustrating it through both simulations and observational applications, the author fills a methodological gap in applied econometrics.

There are some issues that the authors need to address before the manuscript can be considered for publication. The following are my comments describing these issues.

# 1- Contribution and Relevance

- The contribution is methodological and addresses a gap in applied econometrics practice: a formal, reproducible approach for OLS–IV comparison.
- The integration with Oster’s method is original in this context, and the emphasis on implementability (including Stata code) is a major strength.
- The simulations convincingly show that large IV/OLS ratios do not necessarily imply instrument invalidity.

# I would like to sincerely thank the referee for their engaging and constructive comment. Their generous assessment that the paper is "technically solid and methodologically focused" and fills a "methodological gap in applied econometrics" is deeply encouraging. I am also grateful for their recognition of the paper's original integration with Oster’s method and its practical implementability as a "major strength." Their insightful comments have undoubtedly improved the manuscript.

# 2- Theoretical Framework

- The derivation from equations (1)–(5) is correct but dense. Some intermediate economic intuition would help applied readers, e.g., explaining in words: o Why the proportionality assumption is plausible in some settings. o What kinds of omitted variables w are captured by δ in applied terms.
- The paper moves quickly from the univariate to the multivariate setting; a table summarizing symbols, assumptions, and their roles would improve accessibility.

# I am very grateful for this insightful comment. It prompted me to thoroughly revisit the derivations with the specific aim of enhancing their clarity and logical flow. In response, I have restructured the mathematical appendix to create a more intuitive bridge from the univariate to the multivariate case, making the material more accessible without sacrificing rigor. The key steps in this revised derivation are highlighted in red in the latest draft of the manuscript to facilitate your review.3- Assumption Discussion

• The proportional selection assumption (equation (3)) is strong. While the author mentions that prior knowledge of the setting is crucial, more discussion is needed on: o Situations where the assumption is likely violated (e.g., when observables are weak proxies for unobservables).

- Sensitivity of results to mis-specification of Rmax.
- Possible robustness checks (e.g., alternative Rmax values, bounding δ using prior studies).

I have addressed this comment in the main text of the paper. I can expand it as you consider fit.

# 4- Simulation Study

1

• The Monte Carlo section is informative, but the practical meaning of thresholds such as δ<1 or δ<2 is not fully explained. The reader would benefit from a short “practical guidelines” subsection translating these findings into applied advice (e.g., what value of δ should start to raise concern).

Thanks for this comment that I hope improves accesibility to the paper. I have addressed it in a new subsection written ad hoc. I can add any further input you might have.

# 5- Empirical Applications

- **Acemoglu et al. (2001):**
  - The conclusion that no plausible δ can reconcile OLS and IV estimates is striking. The discussion could more explicitly connect this to historical debates on settler mortality and institutions to help contextualize why IV estimates may be inflated.
- **Ciacci (2024):**
  - This example benefits from a clear rationale for the instrument (flights), but robustness to alternative specifications or placebo outcomes would strengthen the case.
  - Since this is the author’s own prior work, a note on possible self-selection in variable choice could be made transparent.

# I thank the referee for their insightful comments on the empirical applications. Regarding the application to Acemoglu et al. (2001), I agree that connecting the finding more explicitly to the historical debates on the settler mortality instrument would be valuable. However, I do not have the setting knowledge to expand that discussion. I seek the referee's guidance on one specific point if the referee considers this necessary.

# The referee suggests testing "robustness to alternative specifications or placebo outcomes" for my 2024 example within the framework of my methodology. I am uncertain how to implement this, as my methodology is designed to assess the gap between a specific OLS and IV estimate from a preferred specification. A placebo test, for instance, would typically aim to show a null effect; comparing a null IV to a null OLS via my method would not be informative for validating the main result. I included the Acemoglu et al. (2001) example as a counterpoint—a seminal case where the method fails to validate the IV estimates because the required delta is implausibly large. I selected my own paper as a contrasting example specifically because its setting offered a clear a priori rationale for the sign of delta which is a prerequisite for the method.

# Therefore, if the referee deems it essential, I am prepared to address this point but would be grateful for further clarification on how the referee envisions incorporating these specific checks into the framework of my paper. I am committed to strengthening the manuscript and am happy to follow the referee's direction on this matter.

# 6- Interpretation and Limitations

• The paper is careful to state that large δ values could indicate either invalid instruments or heterogeneous effects. However:

- A more structured decision tree could help applied researchers interpret results (e.g., “If δ is large, check X, Y, Z…”).
- The policy implications of such findings could be briefly discussed — this would broaden the readership beyond methodologists.

In the subsection created for comment 4 I have also addressed these comments. Please let me know if you believe I should modify them further.

2
