# Supplementary material for: A matter of size: Comparing IV and OLS estimates
Source: PLoS One. 2025 Oct 22;20(10):e0334392. doi: 10.1371/journal.pone.0334392 (PMC12543180; doi:10.1371/journal.pone.0334392)
Supplement: Attachment — Submitted filename: reviewer comments.docx [file pone.0334392.s005.docx]

**A Matter of Size: Comparing IV and OLS estimates**

**Manuscript Number: PONE-D-24-23558**

**Reviewer Comments**

This is a revision of the revised version of the paper which addresses the comments by the reviewers to the first version. I beg to agree with most comments by reviewer #2 and it is my view that the revised version did not address them in a satisfactory manner. *My recommendation is not to publish the paper* on the following grounds:

- There is no doubt that the comparison of OLS and IV estimates is an important issue when someone may be discussing the robustness of estimates of treatment effects.
- The purpose of the article is not clearly defined and the findings in the literature should be acknowledged, in particular Oster (2019). This paper is an implementation of the ideas in Oster (2019) and as such does not add anything new to current knowledge.
- The paper provides examples for the implementation of the second way robustness statements might be made (i.e. making statements about $R_{max}$, (Oster, 2018, p. 196-197). I couldn’t locate the contributions the author claims to have added to the text. If the paper is an application of Oster’s ideas, at least the links with Imbens (2003) and Altonji, Elder & Taber (2005) should be described.
- The issue of evaluating the robustness of results is intrinsically empirical. It is assumed that when designing a study, the researchers make decisions about what is an appropriate size for the treatment effect, how to control for confounders and which covariates should be included. Both theory and experience play an important role at this stage. Some of the confounders and covariates might not be observable at the time the study is being designed, and early decisions are made on how to deal with this problem (i.e. instrumental variables or any other approach). This brings me to the topic of the paper, which is how to assess the magnitude of biases due to unobserved variables and likely measurement errors on the estimation of treatment effects. A well-designed research would determine the size of the proportionality coefficient and the validity of candidates instruments in advance. Common sense suggests that the contribution of instruments to selection should be of a lower magnitude than that from the controls. This leave us with a desired value of 1 for the proportionality coefficient.
- Most evaluations in the literature use quasi-experimental designs or are designed as observational studies. They use instruments as part of an identification strategy to introduce enough variability in the estimation of the treatment effects. Decisions on what a valid set of instruments should be, form part of the design process.
- The paper does not include information on the context in which the Oster´s method is being applied. The point that the validity of instruments is a crucial assumption is something that needed to be stressed early in the introduction.
- Later in the methodological section, there is continuous reference to the fact that the proportionality coefficient may indicate that the instruments might not be valid, a matter that causes confusion when reading the paper.
- Before submission, the paper should have been reviewed by someone whose first language is English. There are lots of language problems.
